# Linguistic Fidelity and Classification Performance of Large Language Models for Generating Synthetic Operative Notes: Evaluation Study

**DOI:** 10.2196/87276

**Published:** 2026-07-03

**Authors:** Meredith Cox, Elaine Lin, Nicholas Oleck, Carlee Jones, Neill Y Li, Suhail K Mithani, Alexander C Allori

**Affiliations:** 1 Division of Plastic, Oral, and Maxillofacial Surgery, Duke University Hospital, 2301 Erwin Road, Durham, NC, 27710, United States, 1 919-668-3110; 2 Department of Orthopaedic Surgery, Duke University Hospital, Durham, NC, United States

**Keywords:** machine learning, natural language processing, large language models, cleft lip, cleft palate, artificial intelligence, AI

## Abstract

**Background:**

Machine learning models for surgical applications require large, diverse datasets; however, data scarcity remains a critical limitation due to privacy regulations, institutional variability, and the rarity of many surgical procedures. Large language models (LLMs) offer a potential solution through synthetic data generation, but their performance and reliability in specialized surgical domains remain underexplored.

**Objective:**

This study aimed to evaluate the linguistic fidelity of LLM-generated operative notes for cleft lip and palate procedures and to assess their impact on natural language processing classifier performance under varying data availability conditions.

**Methods:**

A total of 630 authentic operative notes were obtained from cleft procedures (86 primary cleft lip repairs, 101 primary cleft palate repairs, and 62 primary alveolar bone grafting [ABG] procedures) performed between 2013 and 2024. GPT-4o generated matched synthetic notes using multishot prompting with anonymized examples. Linguistic fidelity was evaluated using BERTScore for semantic similarity, Jensen-Shannon divergence of part-of-speech trigrams for syntactic structure, and Bilingual Evaluation Understudy (BLEU) scores for lexical overlap. Binary classifiers using ClinicalBERT embeddings and logistic regression were trained under both full data and data-scarce (retaining 5% or 10% of positive training cases while retaining the full negative training set) conditions, with and without synthetic augmentation at approximate ratios of synthetic to real notes (1:1, 2:1, 5:1, and 10:1).

**Results:**

Synthetic notes demonstrated high semantic fidelity across all procedures (BERTScore *F*_1_-score: 0.86‐0.88) and low syntactic divergence (Jensen-Shannon divergence: 0.06‐0.08). BLEU scores indicated moderate lexical variation (0.14‐0.19), reflecting distinct but contextually consistent phrasing. With full datasets, synthetic augmentation did not meaningfully affect classifier performance. Under data-scarce conditions retaining 5% of positive training cases while preserving the full negative training set, the area under the curve improved from 0.915 (SD 0.056) to 0.929 (SD 0.033) for cleft lip and from 0.935 (SD 0.036) to 0.949 (SD 0.033) for cleft palate, with smaller gains for ABG (mean 0.983, SD 0.014 to mean 0.987, SD 0.015). When retaining 10% of positive training cases, performance changes were minor across procedures (cleft lip: mean 0.952, SD 0.036 to mean 0.948, SD 0.028; cleft palate: mean 0.945, SD 0.044 to mean 0.956, SD 0.036; and ABG: mean 0.989, SD 0.008 to mean 0.985, SD 0.012).

**Conclusions:**

LLM-generated operative notes exhibit strong semantic and syntactic fidelity to authentic documentation and can enhance model performance in a task-dependent manner when authentic data are limited. These findings suggest that synthetic data generation may address data scarcity challenges in specialized surgical domains, particularly for rare or underrepresented procedures, enabling robust machine learning model development.

## Introduction

Machine learning (ML) and natural language processing (NLP) have emerged as powerful tools for analyzing clinical data, with increasing applications in surgical research and quality improvement initiatives [1-4]. One application of NLP is to analyze unstructured data present in clinical notes such as histories and physical examinations, progress notes, and operative notes. Operative notes represent a particularly rich source of clinical information, containing detailed descriptions of surgical procedures, anatomical findings, and technical variations that can inform patient care and research outcomes. However, developing robust ML models for clinical applications requires large, representative datasets that capture the full spectrum of clinical practice—a requirement that often proves challenging to meet in real-world health care settings [5].

Data scarcity is a significant barrier to ML implementation in clinical settings [6]. This challenge is compounded by regulatory constraints limiting access to patient data, security policies related to protected health information and other sensitive data [7], institutional variations in documentation practices [8], and the inherent class imbalance that occurs in the case of rare conditions or infrequent treatment modalities [9]. When data are scarce, it is difficult, if not impossible, to develop robust ML models in that clinical domain.

Recent advances in generative artificial intelligence, particularly large language models (LLMs) such as GPT-4o (OpenAI), offer a potential solution to clinical data scarcity through *synthetic data generation* [10,11]. LLMs can generate text that mimics the structure and content of authentic clinical documentation. If they are of sufficient quality, synthetic clinical documents such as operative notes could then be used as additional data to train ML models. At the same time, synthetic data may partially reduce reliance on access to identifiable patient records, and the variation in synthetic datasets can simulate diversity in authorship and style of writing, potentially yielding a richer dataset and supporting the development of more generalizable ML models. However, further evaluation is needed regarding the performance of synthetically generated operative notes for ML model training in specialized surgical domains such as cleft and craniofacial care, particularly concerning their semantic fidelity, linguistic authenticity, and impact on downstream classification tasks.

In this proof-of-concept study, we evaluated the ability of LLMs to generate operative notes related to surgical treatment of cleft lip and palate. Cleft lip repair, cleft palate repair, and alveolar bone grafting (ABG) notes were selected for evaluation due to their detailed content, sufficient number of real notes available for comparison, and the likelihood of these procedures being underrepresented in single-institution datasets, making synthetic data generation necessary for augmented training of ML models. In this particular case, the ML model being trained is an NLP classifier of operative procedures.

We hypothesized that artificial intelligence–generated operative notes would exhibit sufficient semantic and syntactic similarity to authentic notes to support classifier training. Our primary objectives were to (1) assess the linguistic quality of LLM-generated notes using standard linguistic metrics for semantic similarity, syntactic structure, and lexical features and (2) evaluate the impact of synthetic data augmentation on NLP classification performance across varying levels of data availability.

## Methods

### Authentic Dataset and Preprocessing

Authentic operative notes from 630 various cleft lip and palate repair procedures were obtained from 311 pediatric patients who underwent cleft and craniofacial procedures between 2013 and 2024. Each note includes multiple procedures, totaling 958 procedures. Of these, 86 notes pertained to primary cleft lip repair, 101 notes pertained to primary cleft palate repair, and 62 notes pertained to primary ABG. The mean patient age was 3.75 (SD 4.1; range 0‐18.75) years. All notes were deidentified prior to analysis in accordance with institutional review board protocols.

Besides the technical report of the operative procedure itself, raw operative notes also include information such as patient demographics, medical history, procedural indications, staffing, equipment, medications or IV fluid, blood loss, and postoperative disposition that could confound classification algorithms with distracting nonprocedural cues. To focus on procedurally relevant content for synthetic note generation, a text preprocessing pipeline isolated target sections within each operative report written by the surgeon. The pipeline used pattern recognition to locate section delimiters and parse notes into discrete segments based on header identification using both direct string matching and approximate matching algorithms to accommodate header variations (eg, “procedure report,” “operative report,” “procedure in detail,” “surgical technique,” and “operative approach”). To enhance parsing accuracy across the heterogeneous note formats, we implemented an optional header normalization module using ML-based deduplication [12] that identified semantically equivalent headers and created standardized mappings.

### LLM Synthetic Data Generation

To compare the linguistic characteristics of synthetic notes to real notes, we generated synthetic operative notes for primary cleft lip repair, primary cleft palate repair, and ABG using GPT-4o. The multishot prompting strategy incorporated multiple examples of authentic, manually anonymized operative notes to guide the model in generating clinically realistic synthetic documentation that preserved the structure, terminology, and narrative flow characteristic of cleft nasolabial repair procedures. An example prompt is shown below:

You are a clinical documentation engine. Write de-identified, realistic operative note for primary lip closure following cleft lip repair. Output only the requested section. Do not invent patient names, dates, hospital names, or MRNs. Maintain internal consistency (laterality, technique, graft type).

Examples:

[Example 1]

[Example 2]

[Example 3]

Using this multishot prompting technique, we generated 86 notes for primary cleft lip repair, 101 notes for cleft palate repair, and 62 notes for ABG procedures to match the number of real notes for each procedure.

**Figure 1.**
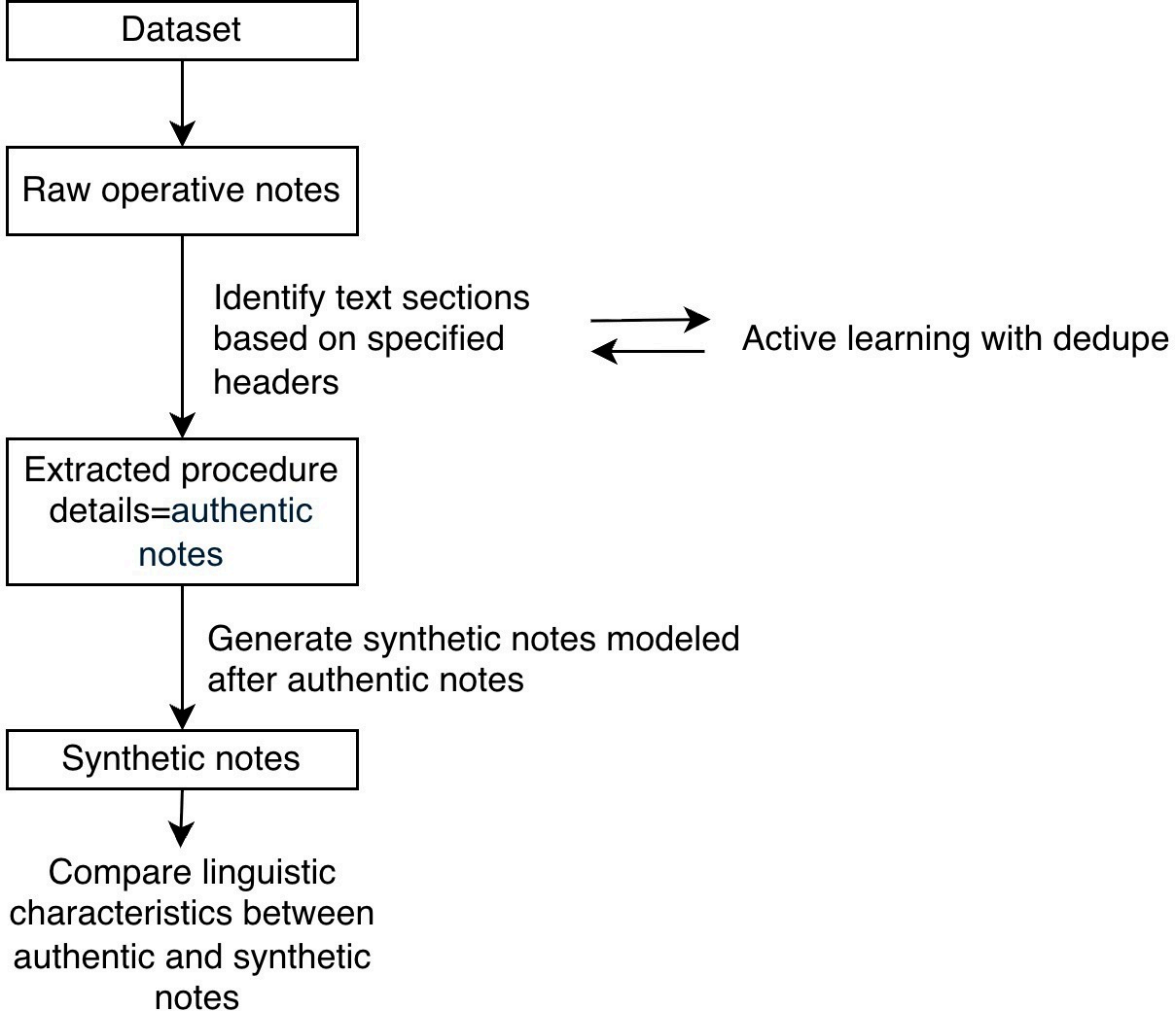
Preprocessing and synthetic data generation steps.

An illustration of this preprocessing and synthetic data generation pipeline is shown in Figure 1.

### Linguistic Assessment of Synthetic Notes

The linguistic fidelity of synthetic notes was evaluated using established metrics described by Chim et al [13]. Specifically, the study describes metrics to evaluate data quality in synthetic texts for tasks such as data augmentation.

Semantic similarity was assessed using BERTScore [14], which measures contextual meaning preservation through deep bidirectional transformer representations. BERTScore metrics were computed using the *bert-score* Python library (version 0.3.13; Python Software Foundation) with a comprehensive pairwise comparison methodology, where each of the authentic notes for each of the 3 procedure types was systematically compared against all synthetic notes to generate precision, recall, and *F*_1_-scores, with reported values representing the mean scores across all possible note pairs.

Syntactic style similarity was quantified using Jensen-Shannon divergence (JSD) of part-of-speech (POS) trigram distributions to capture grammatical structure patterns [15]. This metric quantifies the difference between the distributions of trigrams—sequences of 3 grammatical categories such as adjective, noun, and verb—capturing grammatical pattern frequency, writing style consistency, and syntactic complexity. JSD was computed by extracting POS trigrams from all notes using SpaCy (version 3.8.4; Python Software Foundation) English language model, creating frequency distributions of trigram patterns for authentic and synthetic note collections, and calculating the divergence between these probability distributions using the *scipy.spatial.distance.jensenshannon* function.

Lexical overlap was measured using the Bilingual Evaluation Understudy (BLEU) score, which evaluates n-gram overlap between synthetic and reference texts [16]. These metrics were applied to compare the authentic notes for primary cleft lip repair, primary cleft palate repair, and primary ABG against the synthetically generated notes for each procedure type. The BLEU score was calculated using the *nltk.translate.bleu_score.corpus_bleu* function in the Natural Language Toolkit library (version 3.9.1; Python Software Foundation).

### NLP Classifier Model

A binary classification task was designed to distinguish each primary procedure type from other cleft procedures. For example, primary cleft lip repair would be distinguished from primary cleft palate repair, primary ABG, and other procedures in the dataset. Other cleft procedures include revision lip repairs (14), cleft palate revisions (3), revision ABG (43), velopharyngeal insufficiency repair (32), rhinoplasty (36), auditory procedures (191), suture removal (92), dental rehabilitation (33), oronasal fistula repair (14), cranial procedures (6), orthognathic repositioning (6), and gastrostomy (5). An additional set of 234 less common cases spanned numerous surgical subspecialties, including ophthalmology (strabismus surgery and cataract extraction), neurosurgery (ventriculoperitoneal shunt placement and revision and Chiari decompression), orthopedics (fracture fixation and spinal fusion), general surgery (exploratory laparotomy and bowel resection), urology (cystourethroscopy), and cardiothoracic surgery (congenital heart defect repair). These procedures were performed in patients within the cleft and craniofacial population and were included in the negative class to reflect the full spectrum of procedures encountered in this clinical setting. Each note was labeled based on the presence of the target primary procedure; notes containing multiple procedures were included in the positive class if the target procedure was documented.

Procedure-type classification was selected to assess the utility of synthetic notes in a clinically meaningful context. Beyond serving as a test task, accurate identification of primary procedures is important due to limitations of Current Procedural Terminology coding. For example, the same procedure may be assigned different codes across institutions or surgeons, and some procedures such as ABG lack a dedicated code entirely and are conventionally mapped to adjacent codes. Thus, this classification task is helpful for cohort identification for outcomes research, registry automation, and quality improvement without reliance on Current Procedural Terminology codes.

The dataset was partitioned at the patient level using an 80:20 train-test split to prevent data leakage across splits. All notes from a given patient were assigned exclusively to either the training or test set. Word embeddings were generated using ClinicalBERT, a domain-specific language model pretrained on clinical text. Logistic regression classifiers were trained using these embeddings as input features. To obtain stable performance estimates, all experiments were repeated across 10 random train-test splits with different random seeds. Given the relatively small number of positive cases for each procedure type, performance estimates based on a single fixed train-test split would be highly sensitive to the specific patients assigned to the test set, potentially resulting in overly optimistic or pessimistic estimates of the area under the curve (AUC). Averaging across 10 seeds ensures that reported AUC and accuracy values reflect consistent model behavior across varied data partitions rather than the outcome of any particular split. To assess the effect of augmentation volume under data-scarce conditions, synthetic notes were added at varying synthetic-to-real ratios of 1:1, 2:1, 5:1, and 10:1 relative to the mean number of retained positive training cases per seed.

### Model Evaluation

Model performance was evaluated under three conditions: (1) training on real notes only with the full dataset; (2) training on real notes augmented with synthetic notes using the full dataset; and (3) training under data-scarce conditions retaining only 5% or 10% of positive training cases while retaining the full negative training set, with and without synthetic augmentation. This design enabled assessment of synthetic data utility across different data availability scenarios. Importantly, to isolate the effect of positive case scarcity, data scarcity was applied only to the positive class of the target procedure within the training set—cleft lip, cleft palate, or ABG—while all negative cases and the test set remained unchanged.

Classification performance was assessed using accuracy and AUC. All reported performance metrics (accuracy and AUC) represent mean (SD) values across 10 random train-test splits.

**Figure 2.**
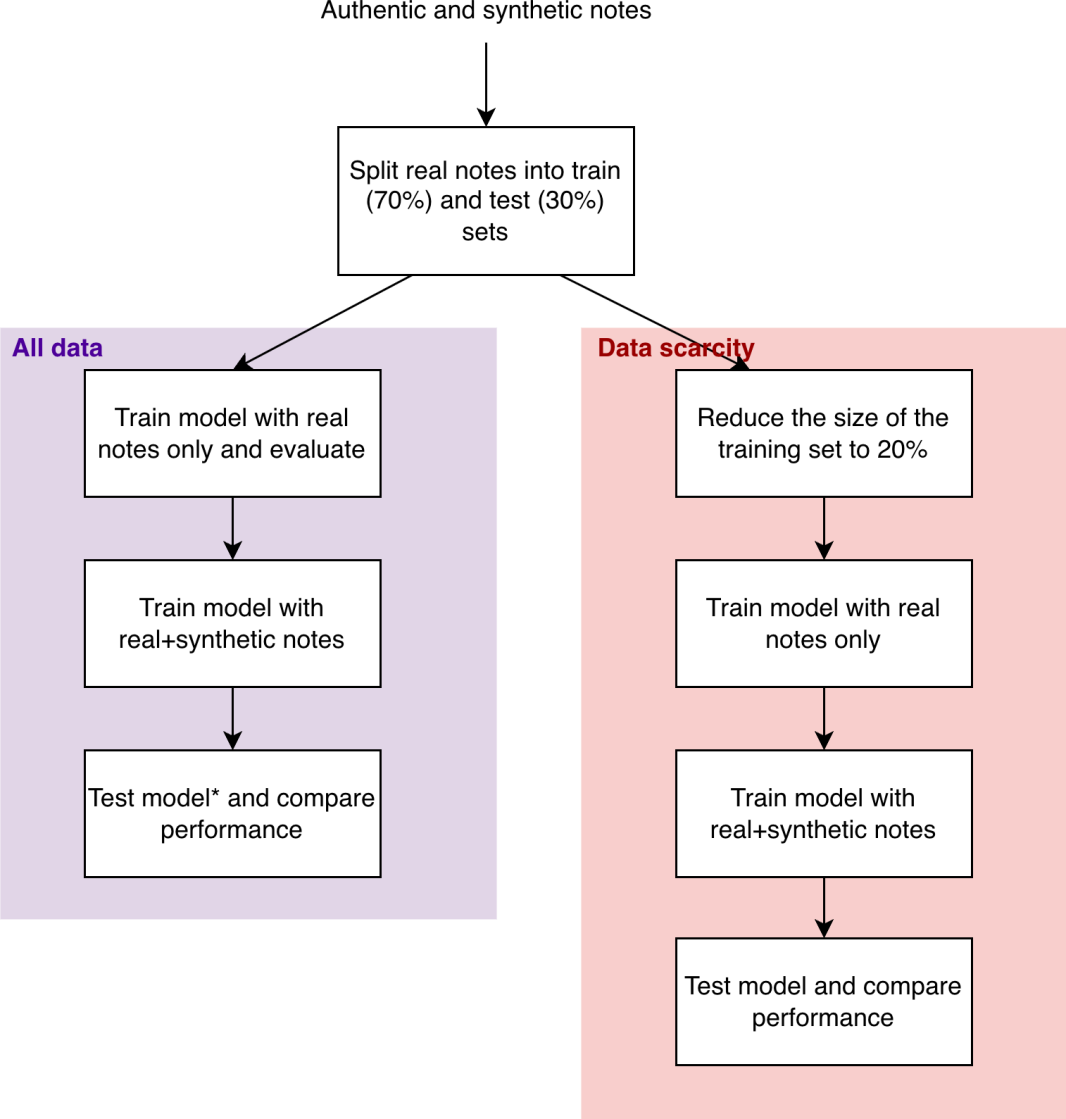
Methodology for evaluating the utility of synthetic notes in a classification task. *The test set consisted exclusively of real notes to assess the impact of synthetic notes on classifier performance in a real-world setting.

Classification model development is illustrated in Figure 2.

### Ethical Considerations

This study obtained institutional review board approval (Pro00104806) for participation in the Allied Cleft & Craniofacial Quality-Improvement and Research Network. Allied Cleft & Craniofacial Quality-Improvement and Research Network member institutions prospectively gather standardized clinical outcomes data aligned with the International Consortium for Health Outcome Measurement’s Standard Set for the Comprehensive Approval of Cleft Care. The network operates as a registered observational cohort study on ClinicalTrials.gov (NCT02702869).

## Results

### Linguistic Analysis of Authentic vs LLM-Derived Synthetic Operative Notes

Across the entire dataset, procedure descriptions had a mean length of 333 (median 325, IQR 5‐1357) words. For primary cleft lip repair procedures specifically, notes averaged 393 (median 343, IQR 23‐890) words. For cleft palate repair procedures, notes averaged 426 (median 407, IQR: 59‐938) words. For ABG procedures, notes averaged 466 (median 462, IQR 276‐937) words.

Representative synthetic operative notes for each procedure type are provided in Multimedia Appendix 1.

Linguistic analysis revealed distinct characteristics of the synthetically generated operative notes across all 3 procedure types. BERTScore analysis showed that synthetic notes retained substantial semantic content compared to real notes across all procedures. For primary cleft lip repair, synthetic notes achieved a precision of 0.86, a recall of 0.87, and an *F*_1_-score of 0.86. Cleft palate repair demonstrated similar performance, with a precision of 0.86, a recall of 0.86, and an *F*_1_-score of 0.86. ABG procedures showed the highest performance, with a precision of 0.88, a recall of 0.88, and an *F*_1_-score of 0.88.

The JSD of POS trigram distributions varied across procedure types, with ABG showing the lowest divergence at 0.06, followed by cleft palate repair at 0.07 and cleft lip repair at 0.08.

In contrast, BLEU scores demonstrated limited lexical overlap between synthetic and real operative notes across all procedures, with notable variation by procedure type. ABG procedures showed the highest lexical overlap, with a BLEU score of 0.19, followed by cleft palate repair at 0.16 and cleft lip repair at 0.14.

Linguistic analysis results are shown in Table 1.

**Table 1.** Linguistic characteristics for synthetic cleft lip, cleft palate, and alveolar bone grafting (ABG) notes.

	BERTScore precision	BERTScore recall	BERTScore *F*_1_-score	JSD^a^	BLEU^b^ score
Cleft lip	0.86	0.87	0.86	0.08	0.14
Cleft palate	0.86	0.86	0.86	0.07	0.16
ABG	0.88	0.88	0.88	0.06	0.19

a JSD: Jensen-Shannon divergence.

b BLEU: Bilingual Evaluation Understudy.

### Surgeon Review

To assess the procedural and anatomic accuracy of notes, a random sample of 10 synthetic notes per procedure type was selected and qualitatively examined by a senior cleft and craniofacial surgeon (ACA). No structured rubric was applied; the reviewer provided open-ended qualitative feedback based on clinical experience. Synthetic notes demonstrated several limitations worth noting. The generated notes appeared to reflect the dictation style of the surgeon whose notes were used as examples, particularly in the level of anatomical detail provided—a style that may not be representative of other surgeons, who may document more tersely (eg, 1 surgeon may document that “the patient had a cleft lip/palate,” while another might document that “the patient had a right unilateral complete cleft lip and alveolus with a Veau-III cleft palate”). The narrative flow was occasionally inconsistent, with some sections being overly verbose, others lacking sufficient detail, and operative steps sometimes appearing slightly out of sequence. On the basis of this informal review, the sampled synthetic notes were judged to be acceptable in quality and clinical content. While this assessment was not blinded or standardized, it provides preliminary face validity for the generated notes within this surgical domain. Moreover, the stylistic variability introduced by these limitations may be viewed as advantageous: notes that are overly brief, loosely organized, or variable in detail may better approximate the range of dictation styles encountered in real-world practice, including notes produced by trainees or time-pressured clinicians.

### ML or NLP Classification Model Performance

#### Performance Using Full Dataset

The test set comprised 20% of authentic notes for each procedure type. As train-test splits were performed at the patient level across 10 random seeds, the number of positive cases in the test set varied across iterations. The mean number of positive test cases was 17.7 (range 11‐25) for cleft lip, 19.7 (range 15‐28) for cleft palate, and 12.7 (range 8‐18) for ABG; reported performance metrics represent averages across all seeds. Synthetic notes were used exclusively for training and were never included in the test set.

When trained on the complete dataset of real operative notes, classification models achieved robust performance across all 3 procedure types. The cleft lip classifier achieved an accuracy of 97.5% (SD 0.7%) and AUC of 0.981 (SD 0.019). Cleft palate classification achieved an accuracy of 96.7% (SD 1%) and AUC of 0.970 (SD 0.014), while ABG procedures showed an accuracy of 97.8% (SD 1.1%) and AUC of 0.991 (SD 0.007). Table 2 summarizes the complete performance metrics for each procedure.

**Table 2.** Classification performance using the full dataset with and without synthetic augmentation.

Procedure type	Test set, n	Real data, mean (SD)		Real+synthetic data, mean (SD)	
		AUC^a^	Accuracy (%)	AUC	Accuracy (%)
Cleft lip	11-25	0.981 (0.019)	97.5 (0.7)	0.977 (0.022)	97.8 (0.9)
Cleft palate	15-28	0.970 (0.014)	96.7 (1)	0.971 (0.018)	97.5 (1.1)
ABG^b^	8-18	0.991 (0.007)	97.8 (1.1)	0.990 (0.006)	97.9 (1)

a AUC: area under the curve.

b ABG: alveolar bone grafting.

The addition of synthetic notes to the full training dataset resulted in minor, clinically negligible decreases in AUC for cleft lip (mean 0.981, SD 0.019 to mean 0.977, SD 0.022) and no meaningful change for cleft palate (mean 0.970, SD 0.014 to mean 0.971, SD 0.018) or ABG (mean 0.991, SD 0.007 to mean 0.990, SD 0.006).

**Figure 3.**
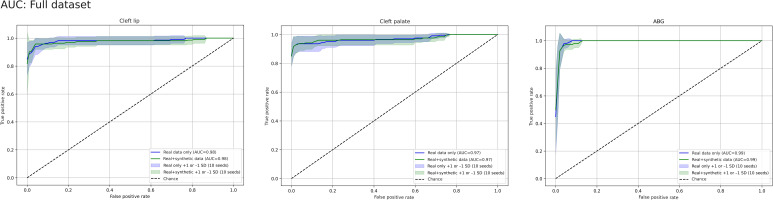
Area under the curves (AUCs) using the full training dataset with and without synthetic augmentation. ABG: alveolar bone grafting.

AUC curves are shown in Figure 3.

#### Performance Under Simulated Data Scarcity

Under data-limited conditions using only 5% or 10% of real positive training cases while retaining the full negative training set, model performance remained relatively high. Using 10% of the data, classifier performance remained strong across all procedure types, with high AUCs (cleft lip: mean 0.952, SD 0.036; cleft palate: mean 0.945, SD 0.044; and ABG: mean 0.989, SD 0.008) and high accuracies (cleft lip: mean 97.3%, SD 1.8%; cleft palate: mean 96.0%, SD 2.1%; and ABG: mean 97.3%, SD 1.4%). When reduced further to 5% of the positive training examples, performance declined modestly, particularly for cleft lip (AUC mean 0.915, SD 0.056) and cleft palate (AUC mean 0.935, SD 0.036), while ABG classification remained robust (AUC mean 0.983, SD 0.014).

**Figure 4.**
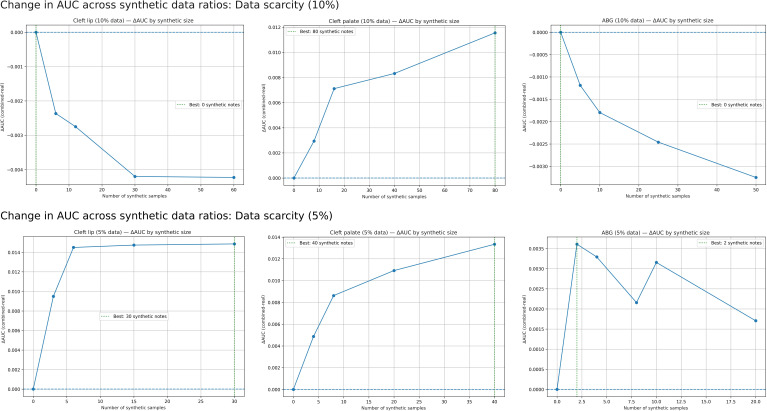
Changes in area under the curve (AUC) across different synthetic-to-real note ratios. ABG: alveolar bone grafting.

**Figure 5.**
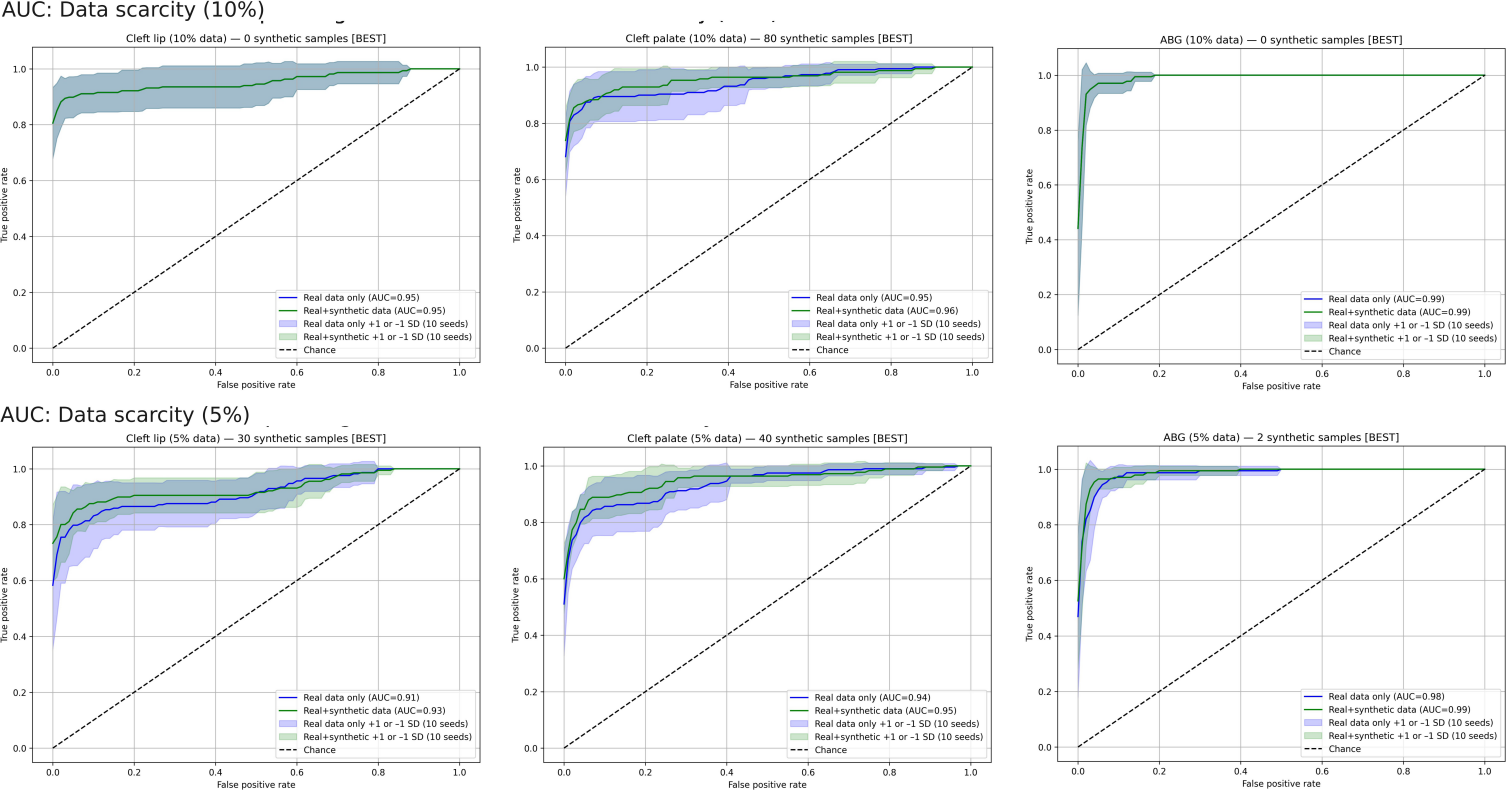
Area under the curves (AUCs) with data-scarce conditions (5% and 10% of training cases retained) with and without synthetic augmentation. The number of synthetic notes yielding the highest mean AUC across 10 random splits is reported for each condition. ABG: alveolar bone grafting.

Incorporation of synthetic notes resulted in minor gains to losses at the 10% data level, with small improvements in AUC for cleft palate repair and minimally decreased AUC for cleft lip repair and ABG. There was little to no change in accuracy across all procedures. In contrast, at the 5% data level, synthetic augmentation provided consistent but modest improvements. For cleft lip classification, AUC increased from mean 0.915 (SD 0.056) to mean 0.929 (SD 0.033), with minor fluctuations in accuracy. Cleft palate classification showed similar gains, with AUC improving from mean 0.935 (SD 0.036) to mean 0.949 (SD 0.033) and minor changes in accuracy. ABG classification demonstrated smaller improvements, with AUC increasing from mean 0.983 (SD 0.014) to mean 0.987 (SD 0.015) and slight gains in accuracy. Tables3  4 present the complete comparative results. Changes in performance as compared to baseline in both data scarcity conditions are shown in Figure 4. AUC curves are shown in Figure 5.

**Table 3.** Classification performance under data-scarce conditions (10% real data) with and without synthetic augmentation.

Synthetic:real note ratio	Cleft lip (test set n≈18), mean (SD)		Cleft palate (test set n≈20), mean (SD)		ABG^a^ (test set n≈13), mean (SD)	
	AUC^b^	Accuracy (%)	AUC	Accuracy (%)	AUC	Accuracy (%)
0:1	*0.952 (0.036)* ^c^	97.3 (1.8)	0.945 (0.044)	96 (2.1)	*0.989 (0.008*)^c^	97.3 (1.4)
1:1	0.950 (0.029)	97.4 (1.7)	0.948 (0.045)	96 (2)	0.987 (0.008)	97.4 (1.2)
2:1	0.949 (0.028)	97.3 (1.6)	0.952 (0.028)	95.6 (2.4)	0.987 (0.008)	97.7 (1.1)
5:1	0.948 (0.028)	97.1 (1.6)	0.953 (0.038)	95.2 (2.1)	0.986 (0.010)	97.4 (1.2)
10:1	0.948 (0.028)	96.4 (1.2)	*0.956 (0.036)^c^*	94.7 (1.8)	0.985 (0.012)	97.1 (1.2)

a ABG: alveolar bone grafting.

b AUC: area under the curve.

c Ratio yielding the highest performance.

**Table 4.** Classification performance under data-scarce conditions (5% real data) with and without synthetic augmentation.

Synthetic:real note ratio	Cleft lip (test set n≈18), mean (SD)		Cleft palate (test set n≈20), mean (SD)		ABG^a^ (test set n≈13), mean (SD)	
	AUC^b^	Accuracy (%)	AUC	Accuracy (%)	AUC	Accuracy (%)
0:1	0.915 (0.056)	94.1 (1.4)	0.935 (0.036)	92.7 (2.1)	0.983 (0.014)	94.8 (1.9)
1:1	0.924 (0.037)	95.2 (2)	0.940 (0.044)	93.3 (1.7)	*0.987 (0.014)^c^*	95.9 (1.7)
2:1	*0.929 (0.035)* ^c^	94.6 (1.8)	0.944 (0.035)	93.7 (2.1)	0.986 (0.014)	96.3 (2.2)
5:1	*0.929 (0.031)^c^*	95.2 (1.8)	0.946 (0.043)	92.7 (1.9)	0.985 (0.015)	96.8 (2.1)
10:1	*0.929 (0.033)^c^*	94.6 (1.8)	*0.949 (0.033)* ^c^	92.5 (1.8)	0.986 (0.015)	96.3 (1.5)

a ABG: alveolar bone grafting.

b AUC: area under the curve.

c Ratio yielding the highest performance.

## Discussion

In this study, we evaluated the characteristics of synthetic operative notes across 3 distinct cleft-related procedures by comparing linguistic characteristics of synthetic operative notes to real operative notes. Moreover, we assessed the utility of synthetic notes in augmenting datasets for training ML or NLP classification models across primary cleft lip repair, cleft palate repair, and ABG procedures.

This experiment showed that LLMs can generate operative notes with high semantic fidelity and preserved syntactic structure. Specifically, the consistently high BERTScores across all 3 procedure types (*F*_1_-scores ranging from 0.86 to 0.88) showed that the synthetically generated notes exhibited strong preservation of clinical meaning and appropriate syntactic structure compared to the authentic operative notes. The synthetically generated notes also showed relatively low JSD values for POS trigrams across all procedures (0.06‐0.08), indicating that syntactic styles (grammatical pattern frequency, writing style consistency, and syntactic complexity) were largely similar between real and synthetic notes, with some variation by procedure complexity.

Interestingly, the synthetic notes showed notable variation in surface-level lexical content across procedure types, as evidenced by the varying BLEU scores (0.14‐0.19). Cleft lip repair showed the lowest lexical overlap (score=0.14), while ABG procedures demonstrated the highest (score=0.19), with cleft palate repair falling between these extremes (score=0.16). This finding suggests that while GPT-4o successfully captured the essential clinical meaning and grammatical structure across all procedure types, it used different vocabulary and phrasing patterns within each procedural category. This lexical divergence may actually be advantageous for data augmentation purposes, as it introduces linguistic variation while preserving clinical content, potentially improving model generalizability by exposing classifiers to diverse ways of expressing similar surgical concepts within different procedure types.

The classification results tentatively suggest that synthetic data generation may be a viable strategy for addressing data scarcity in the cleft and craniofacial domain. When training with the full authentic dataset, synthetic notes offered no measurable improvement in model performance, with AUC values remaining stable or declining negligibly across all procedure types. This suggests that when real data are already sufficient, synthetic augmentation may introduce redundancy or noise rather than additional informative variation. However, under data-scarce conditions (using only 5% or 10% of real positive training cases), synthetic augmentation yielded some performance gains.

When retaining 10% of positive training cases with the full negative training set, the effect of synthetic augmentation was mixed. AUC for cleft lip classification declined minimally across all synthetic note counts (from mean 0.952, SD 0.036 to mean 0.948, SD 0.028 at the highest ratio), suggesting that either the synthetic notes were not helpful or the real-only model was already near its performance limit in this setting. For cleft palate classification, AUC improved incrementally with increasing augmentation, rising from mean 0.945 (SD 0.044) to mean 0.956 (SD 0.036) at the highest synthetic note count. ABG classification remained stable and high throughout (AUC mean 0.989, SD 0.008 to mean 0.985, SD 0.012), with no meaningful benefit from augmentation. Under more severe 5% data scarcity, gains were larger: cleft lip AUC improved from mean 0.915 (SD 0.056) to mean 0.929 (SD 0.033) at 2:1, 5:1, and 10:1 ratios; cleft palate improved from mean 0.935 (SD 0.036) to mean 0.949 (SD 0.033) at a 10:1 ratio; and ABG improved from mean 0.983 (SD 0.014) to mean 0.987 (SD 0.015) at a 1:1 ratio. These results suggest that synthetic augmentation provides greater benefit when real positive training cases are more scarce, with diminishing returns as the availability of real data increases. The consistently high AUC for ABG classification across both scarcity levels and augmentation conditions may reflect a less complex classification task with distinct vocabulary that is inherently less sensitive to training set size. These findings support our expectation that synthetic data can provide value when authentic training examples are limited, though the magnitude of benefit may depend on the inherent difficulty of the classification task and the quality of the synthetic examples.

A key finding of this study is that synthetic augmentation did not substantially degrade classifier performance in the data-scarce conditions and actually provided modest but consistent improvements for cleft lip, cleft palate, and ABG classification at 5% data availability. This pattern suggests that LLM-generated operative notes are noninferior to training on limited real data alone and may offer a practical augmentation strategy when authentic examples are scarce. Even at the lowest augmentation ratios, synthetic notes did not introduce noise sufficient to meaningfully impair classifier performance, which is a meaningful finding given the specialized and technical nature of surgical documentation.

The ability to generate linguistically authentic operative notes has potential implications for clinical research applications. Our findings suggest that synthetic data generation could be explored as a strategy for addressing data scarcity in specialized surgical domains, particularly when certain procedures may be underrepresented in institutional datasets. The semantic preservation demonstrated by high BERTScore values indicates that synthetic notes capture essential clinical information, suggesting they maintain clinical authenticity.

Looking at this research in the broader context of medical artificial intelligence and synthetic data generation, this study contributes to the growing body of research exploring synthetic data as a solution to health care’s persistent data scarcity challenges [17-22], particularly in specialized medical domains where traditional data collection and sharing methods face significant regulatory and practical barriers [23,24]. This builds upon earlier foundational work in medical text generation and augmentation. Traditional data augmentation methods in NLP can be broadly categorized into rule-based, interpolation-based, and model-based approaches. Rule-based techniques apply predefined transformations to existing data such as token-level perturbations (eg, insertion, deletion, and swapping) or syntactic manipulations such as dependency tree modifications. Interpolation-based methods generate new samples by combining inputs and labels from multiple examples, often in embedding or feature space. Model-based approaches use generative models to create paraphrases or entirely synthetic examples that preserve semantic content while introducing linguistic variation [25]. Among model-based approaches, LLMs represent the most recent generative tools, and LLM-based data augmentation approaches are well established for generating pseudo data for augmentation purposes [26].

Researchers in the medical field have increasingly turned to LLMs to address the unique challenges of augmenting existing clinical documentation [27]. One study generated and evaluated synthetic neurosurgical data using GPT-4o, finding that the model could produce high-fidelity tabular data that preserved between-parameter relationships and the direction and strength of correlations [28]. Similarly, LLMs with zero-shot prompting have been shown to generate realistic tabular datasets for perioperative data [11]. Beyond structured data, synthetic text generated by LLMs has been applied successfully to train prediction models for suicidal ideation and depression symptoms [29,30]. Dedicated tools, such as SynthCraft [31], have been developed to support the creation of synthetic medical data more broadly.

The study’s focus on surgical operative notes is an important extension of synthetic data research into specialized clinical domains. While much of the existing literature on medical synthetic data generation has concentrated on more general clinical texts such as progress notes, this study demonstrates the potential for LLMs to capture the nuanced, procedure-specific language and technical terminology that characterizes surgical documentation. Moreover, this study elected to train classifiers of primary cleft lip repair, cleft palate repair, and ABG, discriminating each procedure from other procedures. For example, the primary cleft lip repair classifier distinguished primary cleft lip repair from secondary lip revisions, cleft palate repair, ABG, and cleft rhinoplasty procedures. This is noteworthy because the same patients may undergo multiple procedures in stages over the first 2 decades of life, so the operative notes include many of the same words related to similar anatomy. A successful classifier must therefore identify the correct procedure type despite substantial overlap in vocabulary across related operations. This focus on primary cleft lip procedures is particularly significant because these operations, while foundational to cleft care, exhibit considerable technical variation in surgical approach between surgeons. This makes synthetic data generation especially valuable for developing robust classification models in this domain.

Several limitations should be considered when interpreting these results. First, our evaluation focused on a single surgical domain (cleft and craniofacial procedures) and 1 specific binary classification task per procedure type. The generalizability of these findings to other surgical specialties, more complex procedures, or different NLP tasks remains to be established. Additionally, while our linguistic metrics demonstrated good semantic and syntactic preservation, more comprehensive evaluation of clinical accuracy would be necessary for broader clinical applications.

Another limitation is the 1:1 augmentation design used for synthetic note generation under full data conditions, in which the number of synthetic notes matched the number of real examples for each procedure. While this conservative strategy allowed us to assess the effect of synthetic data without introducing substantial dataset imbalance, it may underestimate the potential benefit of synthetic augmentation when authentic data are abundant. Larger ratios (eg, 5:1 or 10:1) were not tested in this setting due to the computational cost of generating notes at that scale (which would require 860, 1010, and 620 additional notes for lip, palate, and ABG, respectively). However, the absence of any performance improvement even at 1:1 augmentation in a setting where baseline classifiers already achieved AUCs≥0.97 suggests that larger augmentation ratios would be unlikely to provide further benefit. This interpretation is consistent with the data-scarce experiments, where larger augmentation ratios (up to 10:1) were tested and showed diminishing returns as data availability increased. Future work should examine whether large-scale augmentation confers benefit in domains with lower baseline performance or more complex classification tasks where ceiling effects are less likely to obscure potential gains.

An additional limitation relates to model selection and currency. Synthetic notes were generated using GPT-4o; however, different LLMs may exhibit distinct generation characteristics, including differences in verbosity, stylistic consistency, factual reliability, and variability in phrasing. Therefore, the linguistic properties and downstream utility of synthetic operative notes may be model dependent. Although newer models may offer improvements in some domains, they may also introduce different biases or artifacts that affect data augmentation performance [32]. Our findings should be interpreted within the context of the specific model used (GPT-4o). Further work is needed to evaluate how different LLM architectures and versions influence both the quality of generated clinical text and its impact on downstream NLP tasks.

Finally, our evaluation was limited to a single classification task, and the utility of synthetic data may vary depending on task complexity and domain. The singular task assessed here is likely insufficient to draw general conclusions about the utility of synthetic data. Future work should explore alternative generation strategies and downstream tasks, such as different LLM architectures, prompting approaches, and clinical coding applications, to better define the contexts in which synthetic data provide meaningful benefit.

## Supplementary material

10.2196/87276 Multimedia Appendix 1 Representative synthetic operative notes generated by GPT-4o using multishot prompting, covering each procedure type used in model development. Notes were reviewed and approved by a senior cleft and craniofacial surgeon (ACA).

## Abbreviations

ABG: alveolar bone grafting
AUC: area under the curve
BLEU: Bilingual Evaluation Understudy
JSD: Jensen-Shannon divergence
LLM: large language model
ML: machine learning
NLP: natural language processing
POS: part-of-speech
