# Supplementary material for: Linguistic Fidelity and Classification Performance of Large Language Models for Generating Synthetic Operative Notes: Evaluation Study
Source: JMIR Form Res. 2026 Jul 3;10:e87276. doi: 10.2196/87276 (PMC13331248; doi:10.2196/87276)
Supplement: Multimedia Appendix 1 [file formative-v10-e87276-s001.docx]

**Supplementary Appendix A**

The following operative notes were synthetically generated using GPT-4o with a multi-shot prompting strategy, as described in the Methods. Each note represents one procedure type for which synthetic augmentation was used in model development. Notes were reviewed by a senior cleft/craniofacial surgeon (ACA) and rated as satisfactory in quality and content.

**A.1 Cleft Lip Repair — Synthetic Operative Note**

The patient was transported to the operating suite and positioned supine on the table. Following the induction of general anesthesia, the patient's facial region was prepped and draped in conventional sterile fashion. A surgical time-out was undertaken with the full team present to confirm procedure details. Examination re-identified the presence of a left unilateral incomplete cleft lip with associated nasal asymmetry. To address this, markings were delineated following a modified rotation-advancement technique, incorporating a 3mm superior rotation and a 2mm inferior cutaneous triangle. Cartilage reduction at the alar region was planned. A diluted solution of lidocaine with epinephrine was administered for local vasoconstriction‚ ample time was given for its optimal effect. Surgical landmarks were highlighted with methylene blue, and the markings were meticulously scored. Using a #15 scalpel blade, skin incisions were executed, excising the clefted tissue with fine scissors. Dissection ensued to liberate the muscular plane from surrounding skin and mucosal attachments. To facilitate an endonasal rotation, the alar base was sharply separated from the piriform aperture. A Tessier elevator assisted in mobilizing cheek tissues. The anterior caudal septum was carefully dissected from the anterior nasal spine and shifted midline, securing it to the periosteum with 4-0 PDS sutures. Blunt dissection facilitated the mobilization of the medial and lateral crura of the lower lateral cartilage. Oral mucosal closure utilized 4-0 Vicryl sutures, followed by muscular approximation with 5-0 Vicryl, arranged in a manner to simulate the natural philtral ridge. The suture technique ensured optimal alignment of the orbicularis oris muscle over the anterior nasal spine. An alar cinch suture utilizing 5-0 PDS was strategically placed to refine contour. The vermilion triangle was inset into the red line and secured with 6-0 Vicryl and 7-0 chromic sutures. Closure of the nasal sill and floor was achieved using the same suture configuration, with meticulous care to innovate smooth alar base elevation and rotation. A planned 2mm inferior cutaneous triangle was integrated into the medial incision, just superior to the white roll. Slight trimming of the advancement flap allowed for precise insetting with 6-0 Vicryl and 7-0 nylon stitches. Nasal asymmetry correction proceeded through the placement of percutaneous transfixion sutures, adhering to the McComb and Marcus methodologies. This included using 5-0 Monocryl to reform the scroll, optimize interdomal angles, stabilize the lateral crus, and define the alar crease. Upon conclusion of the procedure, the wound was irrigated, thoroughly dried, and protected with Bacitracin. The patient's response to the procedure was favorable, and all surgical counts were verified as correct. The patient recovered from anesthesia without incident and was transported to the post-anesthesia care unit in stable condition.

**A.2 Cleft Palate Repair — Synthetic Operative Note**

The patient was taken to the operating room and placed in the supine position. General anesthesia was induced, and a time-out was conducted. The patient was carefully padded, prepped with standard sterile technique, and draped. A Dingman mouth retractor was placed to ensure unobstructed access to the oral cavity. We infiltrated the cleft with a solution containing lidocaine with epinephrine and waited for the vasoconstrictive effect. Following Bardach's two-flap palatoplasty technique, incisions were made sharply along the cleft margins using a #15 blade. Periosteal elevators were employed to elevate each mucoperiosteal flap. Care was taken to identify the greater palatine vessels; these were preserved by carefully peninsularizing the flaps. Medial fracture of the hamulus was performed bilaterally to enhance palatal mobility. The nasal mucosa was meticulously dissected from the underlying muscle. A multilayered closure was performed, beginning with the nasal mucosa, approximated using 5-0 Vicryl sutures in an inverted manner from posterior to anterior. Attention was then directed toward the dissection of levator veli palatini muscles, releasing them from the hard palate edges. Intravelar veloplasty was undertaken employing a 3-0 Vicryl suture to reapproximate the muscle fibers. The oral mucosa was closed with a combination of 4-0 Vicryl horizontal mattress and 5-0 Vicryl simple sutures for tension-free adaptation. Hemostasis was achieved with careful bipolar cautery. A petrolatum gauze was placed as dressing over the repair. Instrument counts were confirmed correct, and estimated blood loss was minimal. No intraoperative complications occurred. The patient tolerated the procedure well and was transferred to the recovery area in stable condition

**A.3 Alveolar Bone Grafting — Synthetic Operative Note**

The patient was brought to the operating room and placed in a supine position. General endotracheal anesthesia was administered without complications. The surgical sites—oral cavity and left hip—were prepped and draped in a standard sterile fashion. A surgical time-out was conducted with all personnel in attendance. The procedure commenced with the harvest of corticocancellous bone from the left iliac crest. A 4cm incision site was marked, and a solution of lidocaine with epinephrine was infiltrated locally to achieve hemostasis. Allowing adequate time for the anesthetic and vasoconstrictive effects, a #15 blade was used to incise the skin sharply, extending down to the cartilaginous cap of the iliac crest. The cap was incised, and subperichondrial dissection allowed for access to the cancellous bone. An osteotome removed a cortical window, revealing the cancellous bone beneath, which was then harvested using bone curettes. The harvested bone was kept sterile for later use. A hemostatic agent, GelFoam, soaked in local anesthetic, was used to pack the donor site. The cortex was replaced, and the perichondrium, fascia, and dermis were reapproximated with 3-0 and 4-0 vicryl sutures, respectively. Skin closure was achieved with 5-0 monocryl, followed by the application of skin glue, Steri-Strips, and a sterile dressing. Attention was then directed to the oral cavity. The alveolar cleft margins were incised along the gingiva and mucosa. Gingivo-periosteal flaps were elevated on both sides, with the release of dental papillae to facilitate soft tissue closure. Subperiosteal undermining was performed to enhance tissue mobility, and necessary relaxing incisions were made, noting the position of anatomical landmarks such as Stensen's duct. Following full exposure of the osseous margins of the alveolar cleft, including the piriform/nasal walls, the nasal floor mucosa was meticulously separated from underlying scar tissue. Cancellous bone was densely packed into the alveolar cleft up to the level of the piriform rim to support the nasal base. The anterior gingiva-mucosal flaps were then precisely reapproximated and sutured using 4-0 Vicryl, ensuring a watertight closure. The dental papillae were similarly reapproximated. The oral cavity was thoroughly irrigated and suctioned dry. Teeth were cleaned using chlorhexidine mouthwash. An acrylic dental splint was seated appropriately to maintain stability. A soft, flexible orogastric tube was used to clear gastric contents, ensuring the absence of complications. The patient tolerated the procedure well and was extubated without incident. Final counts of instruments and materials were confirmed as correct at the procedure's completion.
